# Supplementary material for: Validation of video analysis of marker-less barbell auto-tracking in weightlifting
Source: PLoS One. 2022 Jan 28;17(1):e0263224. doi: 10.1371/journal.pone.0263224 (PMC8797250; doi:10.1371/journal.pone.0263224)
Supplement: Attachment — Submitted filename: 06_Plo_#2Response-to-reviwers_Ver02.0.doc [file pone.0263224.s002.doc]

# ID: PONE-D-20-38952

Title: Validation of Video Analysis of Marker-Less Barbell Auto-Tracking in Weightlifting

**Response to Reviewer #2**

**Comment 1:**

Page 2, Line 8; Replace period with comma after displacement.

**Response:**

Thank you for pointing this out. We have revised this as suggested.

**Comment 2:**

Page 2, Line 13; The authors should clarify better the practical relevance of their findings. The authors state that 2D-AT could obtain barbell position coordinates with sufficient accuracy to discriminate the difference in barbell velocity due to the lifting performance difference. The meaning of this point is unclear. Are they referring to differences between analysis methods, between exercises or between levels of lifters?

**Response:**

We have revised the sentences which the reviewer pointed out as unclear as follows:

Abstract, Page 2, Lines 13-14

Before: We considered that 2D-AT could obtain barbell position coordinates with sufficient accuracy to discriminate the difference in the barbell velocity due to the lifting performance difference.

After: We considered that 2D-AT could obtain barbell position coordinates with sufficient accuracy to discriminate the difference in the lifter's level and a successful or unsuccessful lift.

**Comment 3:**

Page 3, Line 19; The authors provide data illustrating differences between inferior and superior lifters. The authors should be clear throughout when making such comparisons. Did superior or inferior lifters exhibit more or less horizontal displacement? This section would benefit from a sentence that clarifies technical determinants of successful performance in weightlifting.

**Response:**

Thank you for your comment. We have added some information about inferior and superior lifters and successful performance in weightlifting.

Introduction, Page 3, Lines 18-23:

Added: Previous studies have reported that the barbell backward displacement in the first pull phase was 0.037 m and the peak horizontal velocity in the forward direction was 0.35 m/s larger in superior lifters compared to inferior lifters [1]. Furthermore, the barbell backward displacement after the second pull phase was 0.056 m larger in the inferior lifter than the superior lifter [2]. An analysis of the barbell trajectory shows the difference between successful and unsuccessful lifts, as well as the level of competition in weightlifting.

Introduction, Page 3, Line 24:

Added: Non-national level lifters reported a 0.01-0.02 m decrease in the amount of the horizontal displacement of the barbell before and after the practice of snatch [4]

**Comment 4:**

Page 3, Line 21; The authors state that there was a 0.25 m/s difference in bar linear velocity. Between which groups? Which population exhibited greater velocities?

**Response:**

Based on the reviewer’s comments, we have added details on what variables differed between the groups and how. The order of the references has also been changed.

Introduction, Page 3, Lines 18-21

Before: Previous studies reported a 0.023-0.056 m difference in the amount of horizontal displacement of the barbell during the snatch between an inferior and a superior lifter in national level athletes [1, 2].

After: Previous studies have reported that the barbell backward displacement in 1st pull phase was 0.037 m and the peak horizontal velocity in the forward direction was 0.35 m/s larger in superior lifters compared to inferior lifters [1]. Furthermore, the barbell backward displacement after the seconnd pull phase was 0.056 m larger in the inferior lifter than the superior lifter [2].

Reference, Page 15. and all relevant parts of the manuscript.

[1] → [2], [2] → [1]

**Comment 5:**

Page 3, Line 23; Correct 0.0244 to two decimal places.

**Response:**

The manuscript was revised according to the reviewer’s suggestion.

Introduction, Page 3, Line 24

0.0244 → 0.02

**Comment 6:**

Page 3, Line 25; Why should analysis of barbell trajectory during lifting enhance performance? What is the basis for this?

**Response:**

Since the reference did not clearly include evidence as to why the analysis of barbell trajectory during lifting enhances performance, the text was revised as follows:

Introduction, Page 3, Lines 26-27

Before: Interestingly, the analysis of the kinematic and kinetic variables of the barbell trajectory during the lifting motion in competitions and training, and their application in coaching can aid in improving the weightlifting performance [5].

After: A previous study indicated the need for coaches and scientists to monitor barbell kinematic variables because such variables are correlated with weightlifting performance [5].

**Comment 7:**

Page 3, Line 29; Check wording here. Consider rewording to something like ‘LPTs use a cable attached to a barbell which measures variables using a potentiometer or rotary encoder.’

**Response:**

The manuscript was revised according to the reviewer’s suggestion.

Introduction, Page 3, Lines 29-30

Before: In LPT, a cable is attached to a barbell, and various variables are calculated from a potentiometer or rotary encoder [6].

After: LPTs use a cable attached to a barbell which measures variables using a potentiometer or rotary encoder [6].

**Comment 8:**

Page 3, Line 34; The authors state determinants of success in weightlifting performance. This should be stated earlier in the introduction to give context to discussions on the importance of measuring bar trajectory.

**Response:**

Based on the reviewer's suggestion, a statement about the difference between successful and unsuccessful lifts has been added to the first paragraph of the Introduction. The parts of Line 34 in the original manuscript have not been changed in order to maintain the context of the sentence.

Introduction, Page 3, Lines 21-22

Added: An analysis of the barbell trajectory shows the difference between successful and unsuccessful lifts, as well as the level of competition in weightlifting. Specifically, …

Revised: Furthermore → Specifically

**Comment 9:**

Page 3, Line 39; Change ‘system’ to ‘systems’ and replace ‘is’ with ‘are’.

**Response:**

The manuscript was revised according to the reviewer’s suggestion.

**Comment 10:**

Page 4 Line 49; Replace ‘by’ with ‘when’.

**Response:**

The manuscript was revised according to the reviewer’s suggestion.

**Comment 11:**

Page 4, Line 59; The authors state that “The accuracy was verified by comparing 2D-AT with the gold standard—that is, 3D-MC”. Are they referring to the current study or to previous studies? If referring to previous studies, please include relevant citations. If referring to the current study, please save this information for the Methods section.

**Response:**

The sentences that were pointed out by the reviewer have been moved to the Materials and Methods section. Also, some sentences were deleted to keep the context after the changes.

Introduction, Page 3 → Materials and Methods, Page 6, Line 111

Moved: The accuracy was verified by comparing 2D-AT with the gold standard—that is, 3D-MC.

Deleted: This study aimed to clarify the accuracy of 2D-AT.

**Comment 12:**

Page 4, Line 61 – 68; This detail should be removed from the Introduction and placed in the Methods section.

**Response:**

The sentences that were pointed out by the reviewer have been moved to the Materials and Methods section.

Introduction, Page 3 → Materials and Methods, Page 7, Lines 155-157

Moved: The displacement and velocity in the horizontal and vertical directions, widely used in the biomechanical analysis of snatches, were compared between the methods.

**Comment 13:**

Page 5, Line 77; replace ‘year’ with ‘years’. Since the snatch is a technical lift, the level of participants’ proficiency with this exercise should be clarified for the reader. Did participants have three years of experience in the performance of the snatch? Were participants competitive lifters or did they perform the snatch in training on occasion?

**Response:**

Thank you for your helpful comments. Based on the comments of several reviewers, the sentence has been revised as follows:

Materials and Methods, Page 5, Lines 73-75

Before: The participants had at least 3 year of resistance training and snatch.

After: The participants had at least 3 years of experience in resistance training and the snatch exercise. They were not active weightlifters, but they were weightlifting coaches.

**Comment 14:**

Page 5, Line 79; Please clarify if all participants were informed of the benefits of the study as opposed to just a single participant. Reword this sentence to clarify.

**Response:**

Based on the comments of several reviewers, the sentence has been revised as follows:

Materials and Methods, Page 5, Line 76

Before: The participant was informed…

After: All participants were informed…

**Comment 15:**

Page 5, Line 84; The authors state the barbell used was dedicated for men. Please classify the bar based on its weight and dimensions rather than gender.

**Response:**

Thank you for your helpful comments. Based on the reviewer’s comments, the manuscript has been revised as follows:

Materials and Methods, Page 5, Lines 81-82

Before: A barbell (Eleiko Sport AB, Halmstad, Sweden) dedicated for men and approved by the International Weightlifting Federation (IWF) was used for the experiments.

After: A barbell (Eleiko Sport AB, Halmstad, Sweden) with a length of 2.2 m and a weight of 20 kg, approved by the International Weightlifting Federation (IWF), was used for the experiments.

**Comment 16 and 17:**

Page 5, Line 88; Greater clarity required here. How many repetitions did participants perform at each load? How much recovery did participants receive between repetitions within and between loads? Why did the authors choose the loads presented? Considering the intended application of the current data to competition and the use of maximum loads during competition, is the data valid under one-repetition maximum conditions? Please clarify.

Page 5, Line 89; What is meant by the “The warming-up method and the rest time between lifting were free”? Did participants complete their own desired warm-up routine? Please reword.

**Response:**

We apologize for the lack of clarity in the sentence of the manuscript. Criteria for selecting loads for experiments and rest methods have been added and revised to the manuscript as follows:

Materials and Methods, Page 5, Lines 87-90

Added: Preliminary experiments showed that a load of less than 50% of one-repetition maximum would result in an unnatural snatch motion due to low-load, therefore it was excluded from the measurement conditions. In addition, the experiment with the maximum lifting weight was not employed to avoid risks. In the experiment, a maximum of five consecutive snatches was allowed.

Materials and Methods, Page 5, Lines 90-91

Before: The warming-up method and the rest time between lifting were free.

After: The warming-up method and the rest time between lifting were decided by each participant.

**Comment 18:**

Page 6, Line 102; The authors state that two reflective markers were placed at the end of the barbell. This information is also stated in line 93. Please clarify how many markers were attached the barbell and digitized? This should be stated once only.

**Response:**

Thank you for your helpful comments. Based on the reviewer’s comments, the manuscript has been revised as follows:

Materials and Methods, Page 5, Lines 94-95

Before: Two reflective markers were placed at the barbell’s right end (Fig 1).

After: Two reflective markers were placed at each of the barbell’s right and left ends, resulting in a total of four markers (Fig 1).

Materials and Methods, Page 6, Lines 104-107

Deleted: … two reflective markers recorded at 600 Hz were also attached to the barbell’s left end.

Added: …the length of the barbell was calculated from the left and right points of the barbell recorded by the 3D-MC system. The distance between the midpoint of the two markers on the right end of the barbell and the midpoint of the two markers on the left end of the barbell was taken as the length of the barbell.

Materials and Methods, Page 6, Lines 107-108

Before: The mean difference in the distance between the left and right markers (barbell length) against 2,200 mm…

After: The mean difference in the barbell length against 2.2 m…

**Comment 19:**

Page 6, Line 103; It is stated that barbell length was regulated for competition. Please clarify the location of data collection. Specifically, were data collected in a lab or at a competition venue?

**Response:**

In accordance with the revision of Comment 15, the part pointed out by the reviewer above has been deleted from the manuscript because its contents were overlapping. In addition, a sentence has been added about the place (laboratory) where the measurement was performed.

Materials and Methods, Page 6, Lines 107

Deleted: The length of the barbell for the competition was regulated at 2,200 mm.

Materials and Methods, Page 6, Lines 119-120

Added: All experiments were conducted in the laboratory, not at the competition venue.

**Comment 20:**

Page 6, Line 104; Change 2,200 mm to 2.2-m.

**Comment 21:**

Page 6, Line 113; Insert ‘the’ before ‘weightlifting platform’

**Comment 22:**

Page 7, Line 135; Insert ‘was’ after ‘bar’.

**Comment 23:**

Page 7, Line 144; Insert ‘the’ after ‘of.

**Comment 24:**

Page 8, Line 167; Insert ‘the’ before ‘snatch’.

**Comment 25 and 26:**

Page 8, Line 168 and 169; Replace ‘participant’ with ‘participants’.

**Response:**

The manuscript was revised according to the reviewer’s suggestions in Comments 20-26.

**Comment 27:**

Page 8, Line 183; The authors present ICC data describing the level of agreement between the two methods. Please include also the 95% confidence interval of the ICC to identify the upper and lower bound intervals that describe the ICC.

**Response:**

Thank you for your helpful comments. We have added the 95% confidence interval of the ICCs.

Results, Page 9, Lines 189-191

Added: ICC (DxF: 0.997, 95%CI 0.994–0.998, DxB: 0.993, 95%CI 0.990–0.995, DyMH: 0.999, 95%CI 0.999–0.999, pVxF: 0.944, 95%CI 0. 894–0. 967, pVxB: 0.995, 95%CI 0.987–0.997, pVy: 0.995, 95%CI 0.993–0.997).

**Comment 28:**

Page 9, Line 196; The authors state that “pVxF showed a relatively larger value than pVxB and pVy”. Please simplify this statement to enhance clarity for the reader. Is it meant that the 2D-AT method exhibited greater error for velocity measures relative to the 3D-MC method?

**Response:**

Thank you for your constructive comment. According to reviewer’s comments, a description of the precision of pVxF has been added to the manuscript.

Results, Page 9, Lines 203-204

Added: This indicates that the precision of pVxF is lower than that of pVxB and pVy within the 2D-AT method.

**Comment 29:**

Page 9, Line 199; Please insert a sentence that clarifies the specific nature of the limit-of-agreement to suggest that data showed good agreement between the 2D-AT and 3D-MC methods for kinematic measures.

**Response:**

Thank you for your constructive comment. According to the reviewer’s comments, a description of the LoA has been added to the manuscript.

Results, Page 9, Lines 207-209

Added: The limits of agreement are for visual judgement of how well two methods of measurement agree. The smaller the range between these two limits the better the agreement is.

**Comment 30:**

Page 10, Line 218; Delete “measures obtained by”.

**Response:**

The manuscript was revised according to the reviewer’s suggestion.

**Comment 31:**

Page 10, Line 225; What is meant by having a few restrictions on tools and places? Is it meant that the 2D-AT method can be used with limited equipment in a variety of environmental settings?

**Response:**

Thank you for your constructive comment. In order to avoid ambiguity in the sentences, the manuscript was revised as follows:

Discussion, Pages 10-11, Lines 230-234

Before: It was considered that by employing the 2D-AT system, it was possible to analyze the barbell trajectory with high accuracy, low cost, and with only a few restrictions on tools and places.

After: For weightlifting analysis, the 3D-MC system is costly and requires a large space to place many cameras. It was considered that by employing the 2D-AT system that needs only one digital camera, it was possible to obtain the barbell trajectory at a lower cost than the 3D-MC system, and without the limitations of location, while maintaining the accuracy of the measurement.

**Comment 32:**

Page 10, Line 233; The meaning of this sentence is unclear. Consider rewording.

**Response:**

The sentences have been revised for clarity as follows:

Discussion, Page 11, Lines 240-243

Before: The magnitude of this bias is considered to be sufficiently smaller than the corresponding of the variable difference indicating the snatch technique, which has been clarified in the previous study.

After: The bias of the variables for barbell displacement presented in this study was smaller than the differences in barbell variables between athletes with different lifting performances and between successful and unsuccessful snatches presented in previous studies.

**Comment 33:**

Page 10, Line 236; Check wording here. Are the authors referring to differences between successful and unsuccessful lifts?

**Response:**

In order to avoid ambiguity in the sentences, the manuscript was revised as follows:

Discussion, Page 11, Lines 245-246

Before: …with different performances.

After: …with differences between the performance level of lifting and success or failure.

**Comment 34:**

Page 11, Line 239; The authors are urged to be specific throughout to aid clarity. What is meant by high and low competition levels?

**Response:**

For the reader who is not so knowledgeable about weightlifting, the manuscript has been revised as follows:

Discussion, Page 11, Lines 247-249

Before: …previous studies reported that the pVxF (barbell maximum forward velocity) was significantly lower by 0.25 m/s in athletes with high than in those with low competition levels [2].

After: … it was shown that the value of pVxF (barbell maximum forward velocity) was significantly lower by 0.35 m/s in the lifter who lifted a higher weight in the competition than the lifter who lifted a lower weight [1].

**Comment 35:**

Page 11, Line 248; The meaning of this sentence is unclear. Consider rewording.

**Response:**

Due to other revisions that increased the word count of the manuscript, unclear sentences that had been pointed out as low priority were deleted from the manuscript.

Discussion, Page 11, Line 260

Deleted: As multiple loads of snatches have been analyzed and samples of variables were widely obtained, the results of this study could be applied even if a lifting technique were to be developed in the future.

**Comment 36:**

Page 11, Line 255; Insert ‘magnitude of’ before pVxF and delete ‘value magnitude’.

**Response:**

The manuscript was revised according to the reviewer’s suggestion.

**Comment 37:**

Page 12, Line 267; Please change the reference to ‘women’s barbells’ to ‘barbells used during women’s competitions’. Please also refer to ‘barbell’s used during men’s competitions’.

**Response:**

The manuscript was revised according to the reviewer’s suggestion as follows:

Discussion, Page 12, Lines 277-279

Before: In particular, women's barbells for competition almost always have a yellow end. This is a lower contrast than the dark blue (men's barbells for competition).

After: In particular, barbells used during women’s competitions almost always have a yellow end. This is a lower contrast than the barbell used during men’s competitions that was employed in this study, which has a dark blue end.
