# Supplementary material for: Validation of video analysis of marker-less barbell auto-tracking in weightlifting
Source: PLoS One. 2022 Jan 28;17(1):e0263224. doi: 10.1371/journal.pone.0263224 (PMC8797250; doi:10.1371/journal.pone.0263224)
Supplement: Attachment — Submitted filename: 33_Plo_2Response-to-reviwers_Ver02.0.doc [file pone.0263224.s003.doc]

# ID: PONE-D-20-38952R1

Title: Validation of video analysis of marker-less barbell auto-tracking in weightlifting

**Response to Reviewer #2**

**Comment 1:**

Page 5, Line 86; check wording of this sentence. Is it true that participants performed 160 snatches at each load? The authors should reword to clarify the specific number of repetitions completed by each participant at each load.

**Response:**

We thank the reviewer for this pertinent suggestion. We apologize for the inadequacies of the text, as pointed out by the reviewers. We have revised the manuscript accordingly:

Materials and Methods, Page 5, Lines 87–88

Before: A total of 160 snatches were recorded 5 times for each load.

After: A total of 160 snatches were recorded, whereas every subject completed five repetitions for each load condition.

**Comment 2:**

Page 5, Line 93; insert ‘system’ after 3D-MC.

**Response:**

We thank the reviewer for this constructive comment. We have revised the manuscript following your comment.

Materials and Methods, Page 5, Line 94

Before: … 3D-MC (Vicon MX; Vicon Motion Systems, Oxford, UK).

After: … 3D-MC system (Vicon MX; Vicon Motion Systems, Oxford, UK).

**Comment 3:**

Page 5, Line 97; the authors state that barbell position was measured at “a higher sampling frequency”. Please clarify the exact sampling frequency to aid repeatability of the study.

**Response:**

We thank the reviewer for pointing this out. We have revised the sentences accordingly:

Materials and Methods, Pages 5–6, Lines 99–100

Before:

Therefore, the barbell position was measured at a higher sampling frequency to accurately measure the trajectory of the barbell including the vibration due to the collision.

After:

Therefore, the barbell position was measured at a higher sampling frequency in the present study (600 Hz) than those in the previous studies (200–250 Hz) to accurately measure the trajectory of the barbell, including the vibration due to the collision.

**Comment 4:**

Page 5, Line 113; insert ‘at a’ before ‘sampling’. Insert ‘using a’ before ‘shutter’. Reword ‘and white balance’ with ‘using a white balance setting’.

**Response:**

We thank the reviewer for this pertinent comment. We have revised the manuscript according to your comment.

Materials and Methods, Page 6, Lines 115–116

Before: … sampling rate of 100 Hz, shutter speed of 1/500 s, and white balance of 5500 K.

After: … at a sampling rate of 100 Hz, using a shutter speed of 1/500 s, and using a white balance setting of 5500 K.

**Comment 5:**

Page 9, Line 203; The authors state that “pVxF showed a relatively larger value than pVxB and pVy”. Please simplify this statement to enhance clarity for the reader. Is it meant that the 2D-AT method exhibited greater error for velocity measures relative to the 3D-MC method? This has not been addressed since the original review.

**Response:**

We thank the reviewer for this very important suggestion. We have revised the manuscript following your suggestion.

Materials and Methods, Page 9, Lines 205–207

Before:

… however, among the velocity-related variables, pVxF showed a relatively larger value than pVxB and pVy. This indicates that the precision of pVxF is lower than that of pVxB and pVy within the 2D-AT method.

After:

… however, among the velocity-related variables, pVxF showed a relatively larger value than pVxB and pVy. This indicates that pVxF has a relatively larger statistical variability in the measurement results among the variables of velocity in the 2D-AT method.

**Comment 6:**

Page 11, Line 242; the authors should be more concise. Consider rewording to something like ‘Therefore, it is considered that 2D-AT can obtain the barbell position coordinates with sufficient accuracy to quantify differences in the barbell displacement between successful and unsuccessful lifts as well as between different levels of athlete’.

**Response:**

We thank the reviewer for this valuable comment. We have revised the manuscript following your comment.

Discussion, Page 11, Lines 246–247

Before:

Therefore, it is considered that 2D-AT can obtain the barbell position coordinates with sufficient accuracy to distinguish the characteristic of the difference in the barbell displacement amount between liftings with differences between the performance level of lifting and success or failure.

After:

Therefore, it is considered that 2D-AT can obtain the barbell position coordinates with sufficient accuracy to quantify differences in the barbell displacement between successful and unsuccessful lifts as well as between different levels of lifters.

＊

The manuscript was checked by a native English speaker, and some revisions were made to the entire manuscript, such as with regard to articles ("the") and comma (", ").
